# The Biosafety Research Road Map: The Search for Evidence to Support Practices in Human and Veterinary Laboratories

**DOI:** 10.1089/apb.2022.0040

**Published:** 2023-06-05

**Authors:** Stuart D. Blacksell, Sandhya Dhawan, Marina Kusumoto, Kim Khanh Le, Kathrin Summermatter, Joseph O'Keefe, Joseph Kozlovac, Salama Suhail Almuhairi, Indrawati Sendow, Christina M. Scheel, Anthony Ahumibe, Zibusiso M. Masuku, Allan M. Bennett, Kazunobu Kojima, David R. Harper, Keith Hamilton

**Affiliations:** ^1^ Mahidol-Oxford Tropical Research Medicine Unit, Faculty of Tropical Medicine, Mahidol University, Bangkok, Thailand.; ^2^ Centre for Tropical Medicine and Global Health, Nuffield Department of Medicine, Nuffield Department of Medicine Research Building, University of Oxford, Oxford, United Kingdom.; ^3^ Institute for Infectious Diseases, University of Bern, Bern, Switzerland.; ^4^ Ministry for Primary Industries, Wellington, New Zealand.; ^5^ United States Department of Agriculture, Agricultural Research Service, Beltsville, Maryland, USA.; ^6^ Abu Dhabi Agriculture and Food Safety Authority, Abu Dhabi, United Arab Emirates.; ^7^ Research Center for Veterinary Science, National Research and Innovation Agency, Indonesia.; ^8^ WHO Collaborating Center for Biosafety and Biosecurity, Office of the Associate Director for Laboratory Science, Center for Global Health, Centers for Disease Control and Prevention, Atlanta, Georgia, USA.; ^9^ Nigeria Centre for Disease Control and Prevention, Abuja, Nigeria.; ^10^ National Institute for Communicable Diseases of the National Health Laboratory Services, Johannesburg, South Africa.; ^11^ UK Health Security Agency, Porton Down, Salisbury, United Kingdom.; ^12^ Department of Epidemic and Pandemic Preparedness and Prevention, World Health Organization (WHO), Geneva, Switzerland.; ^13^ The Royal Institute of International Affairs, London, United Kingdom.; ^14^ World Organisation for Animal Health (OIE), Paris, France.

**Keywords:** miscellaneous pathogens, respiratory pathogens, bioterrorism/zoonotic pathogens, viral hemorrhagic fevers, biosafety gap

## Abstract

**Introduction:**

Lack of evidence-based information regarding potential biological risks can result in inappropriate or excessive biosafety and biosecurity risk-reduction strategies. This can cause unnecessary damage and loss to the physical facilities, physical and psychological well-being of laboratory staff, and community trust. A technical working group from the World Organization for Animal Health (WOAH, formerly OIE), World Health Organization (WHO), and Chatham House collaborated on the Biosafety Research Roadmap (BRM) project. The goal of the BRM is the sustainable implementation of evidence-based biorisk management of laboratory activities, particularly in low-resource settings, and the identification of gaps in the current biosafety and biosecurity knowledge base.

**Methods:**

A literature search was conducted for the basis of laboratory design and practices for four selected high-priority subgroups of pathogenic agents. Potential gaps in biosafety were focused on five main sections, including the route of inoculation/modes of transmission, infectious dose, laboratory-acquired infections, containment releases, and disinfection and decontamination strategies. Categories representing miscellaneous, respiratory, bioterrorism/zoonotic, and viral hemorrhagic fever pathogens were created within each group were selected for review.

**Results:**

Information sheets on the pathogens were developed. Critical gaps in the evidence base for safe sustainable biorisk management were identified.

**Conclusion:**

The gap analysis identified areas of applied biosafety research required to support the safety, and the sustainability, of global research programs. Improving the data available for biorisk management decisions for research with high-priority pathogens will contribute significantly to the improvement and development of appropriate and necessary biosafety, biocontainment and biosecurity strategies for each agent.

## Introduction

Preliminary study and discussion among biosafety professionals suggest that the current evidence base to inform laboratory biological risk management has gaps and that biosafety and biosecurity policies are not always based on clear evidence. Gaps in the evidence base and nonevidence-based approaches may result in the use of unsound or unnecessary biosafety procedures. This can increase costs and create challenges regarding laboratories' sustainability. For example, maintaining highly sophisticated equipment may increase risks with little or no increment in safe and secure operations. Unnecessary measures can overburden, distract, or demotivate the laboratory worker. Furthermore, unsound procedures may give the laboratory worker a false sense of security while providing questionable levels of protection.

The World Organization for Animal Health (WOAH, formerly the OIE), World Health Organization (WHO), and Chatham House collaborated to improve the sustainable implementation of laboratory biological risk management, particularly in low-resource settings. This study involved assessing the current evidence base required for laboratory biological risk management performance to provide better access to evidence, identify research and capability gaps that need to be addressed, and provide recommendations on how an evidence-based approach can support biosafety and biosecurity in low-resource settings.

The Biosafety Research Roadmap (BRM) project aims to support the application of laboratory biological risk management and improve laboratory sustainability by providing an evidence base for biosafety measures (including engineering controls) and evidence-based biosafety options for low-resource settings. This will inform strategic global health security decisions and laboratory system investments.

## Methodology

### Technical Working Group

The BRM project involved the formation of a 15-member technical working group (TWG) that was interdisciplinary, inclusive of researchers from public health, clinical science, biomedical science, epidemiology, and veterinary science, as well as experts in the fields of infectious diseases, biosafety and biosecurity, animal health, and disease control and prevention.

### Objectives of the BRM

The objectives of the BRM were to perform a gap analysis for a selected list of priority pathogens on procedures related to diagnostic testing and associated research for those pathogens, including but not limited to sample processing, testing, animal models, tissue processing, necropsy, culture, storage, waste disposal, and decontamination.

The TWG was asked to determine the sufficiency and identify gaps (1) in scientific evidence to perform a biological risk assessment for the specified priority pathogens and (2) to support the biosafety practices commonly used while handling the specified priority pathogens. Furthermore, the TWG was to examine commonly used biosafety practices to determine their suitability and sustainable feasibility to implement in a low-resource setting and identify alternative evidence-based measures more suitable for low-resource settings.

### Selection of the Priority Pathogens

A final priority pathogen list included—Group A: miscellaneous pathogens: foot and mouth disease and *Shigella* spp.; Group B: respiratory pathogens: *Mycobacterium tuberculosis* and zoonotic avian influenza, and SARS-CoV-2; Group C: bioterrorism/zoonotic pathogens: *Bacillus anthracis* and *Brucella melitensis*; Group D: viral hemorrhagic fevers: Crimean Congo hemorrhagic fever and Lassa fever.

### Research Strategy

TWG members were assigned to each pathogen group depending on their area of expertise to determine the evidence, knowledge gaps, and analysis of the findings for each pathogen.

Initial literature searches were performed for each pathogen in the approved pathogen list. The team screened databases, websites, publications, reviews, articles, and reference libraries for relevant data. Searches were conducted using related search terms to find the evidence and knowledge gaps in the biosafety of the chosen pathogens. The main research domains used to perform the literature searches were the ABSA International (ABSA) database, Belgian Biosafety Server, US centers for disease control and prevention reports, WHO reports, PubMed, and internet searches for terms related to biosafety matters, including, for example, inactivation, decontamination, laboratory-acquired infections (LAIs), laboratory releases, and modes of transmission.

Where necessary, working group members were approached for their expertise and other relevant information on specific subject areas. The information collated aimed to include the most recent studies and data to ensure the assessment was up to date. However, earlier studies were cited in cases where recent publications were challenging to find.

The findings of the literature searches were compiled into preliminary biosafety sheets comprising pathogen classification, pathogen characteristics, clinical and laboratory hazard identification, risk factor assessment, an overview of the evidence, and potential gaps in biosafety (with direct evidence quotes). The summary of evidence and potential gaps in biosafety was divided into five main sections: route of inoculation/modes of transmission, infectious dose, LAIs, containment releases, and disinfection and decontamination strategies.

## Results and Discussion

### Evidence Gaps for Specific Pathogens

The authors have identified a series of evidence gaps for the specific pathogens that were studied. The main topic areas where gaps are described include decontamination and inactivation, LAI, engineering controls, transmission route under experimental conditions, organizational measures, risk assessment, infectious dose, personal protective equipment (PPE) use, measures in diagnostic settings, and occupational health and surveillance measures. Table 1 provides a comparative overview of the gap analysis described for each pathogen. The pathogen-specific articles provide references, and additional granularity regarding specific gaps and how they should be addressed.

**Table 1.** Identified biosafety gaps in specific pathogens

Group	Pathogen	Gap 1	Gap 2	Gap 3	Gap 4	Gap 5	Gap 6
Miscellaneous pathogens	*Shigella* spp. and enteric pathogens	Concentrations and optimal contact times of common disinfectants such as sodium hypochlorite, ethanol, glutaraldehyde, iodine, phenolics, and formaldehyde.	*Shigella* spp. LAIs likely to be under-reported				
	FMD	Requirement for a shower when leaving research laboratories	Transmission route under experimental conditions	Justification for 3-day quarantine rule after working with FMD	Optimized fumigation parameters for a range of chemicals	Analysis of the advantages and disadvantages of each certain technical measure to apply suitablely for different laboratories	Establish a risk assessment framework to determine the risk mitigation measures for each laboratory
Respiratory pathogens	Zoonotic avian influenza virus (H5N1)	No known laboratory-associated infections	Infectious dose in humans also remains unknown.	Appropriate concentration and contact times for a number of common disinfectants.			
	SARS-CoV-2	Infectious dose	PPE when using rapid diagnostic tests	Laboratory-acquired infections			
	*Mycobacterium tuberculosis*	Primary containment using BSC class 1 or BSC class 2					
Zoonosis	*Bacillus anthracis*	Infectious dose for cutaneous and gastrointestinal infections is unknown.	Most appropriate PPE and contaminated material disposal procedures during and after necropsies in low resource settings				
	*Brucella melitensis*	Working concentrations for many of the chemical disinfectants, including sodium hypochlorite, aldehydes, iodophors, halogens, and quaternary ammonium compound.					
Viral hemorrhagic fevers	CCHFV	Safe handling of CCHFV-infected ticks	Infectious dose of the agent in the tick bite to cause an infection.	Safe collection and handling protocols			
	Lassa virus	Cutaneous and gastrointestinal infections dose.	Working with samples outside infectious disease areas, that is, cross-matching for transfusion of specimen transportation	The role asymptomatic virus carriers (estimated to be 25%) play in contributing to the cyclic/seasonal propagation of the virus in endemic communications.			

BSC, biological safety cabinet; CCHFV, Crimean Congo hemorrhagic fever virus; FMD, foot and mouth disease; LAI, laboratory-acquired infection; PPE, personal protective equipment.

### Suggested Requirements for Future Applied Biosafety Research

Based on the outcomes from the WOAH BRM, there is a requirement to perform applied biosafety research to determine the evidence base for biosafety and biorisk practices. The only way for staff to be confident in biosafety practices is to understand the basis on which activities are decided rather than the perpetuation of biosafety “folklore.” The following is a list of questions that need to be addressed to provide the basis for a full biosafety, biocontainment, and biosecurity evidence base. These questions need to be addressed to provide the basis for a complete biosafety, biocontainment, and biosecurity evidence base. Often procedures are performed without sufficient forethought because “it's always been done this way.”

### Applied Biosafety Research Priorities

A complete list of applied biosafety research priorities is presented in Table 2 and can be categorized as engineering, pathogen-based issues, decontamination disinfection and activation, and diagnostic and research issues.

**Table 2.** Applied biosafety priorities

Applied biosafety priorities	
Engineering	
Biological safety cabinets	• Optimal frequency from recertification of biosafety cabinets and HVAC HEPA filters. • Requirement to decontaminate through gaseous fumigation biosafety cabinets before recertification or other related procedures?
Facility biocontainment issues	• Standardization of leak rate measurement/air tightness. • Impact of unscheduled laboratory HVAC system shutdown on biocontainment • Impact of aging on the integrity of biocontainment facilities
New technologies risks	• Risks posed by new equipment that generate aerosols
Showering out of high-containment facilities	• Rationale for showering out of some facilities where the laboratory is not the primary containment (i.e., nonanimal rooms)
PPE	• Correct order to remove PPE depending on activity • Reuse and/or decontamination single/multiple use PPE such as N95 respirators, gloves, and coveralls
Pathogen-based issues	
Transmission	• Evidence of infectious transmission on fomites
	• Aerosol production during certain lab procedures (e.g., use of point of care testing system)
Decontamination, disinfection, and inactivation	
Standardized decontamination and disinfection recommendations.	• Standardized decontamination and disinfection recommendation based on scientific evidence against a range of pathogens.
Utility of gaseous decontamination	• Determine efficacy of vapor phase decontamination against various different infectious agents?
DNA/RNA extraction	• Determine efficacy of various methods for DNA/RNA extraction to inactivate infectious agents in clinical samples
Diagnostic and research issues	
DBS	• Risks associated with sample transport of DBS?
	• Risks associated with long-term storage and punch collections from DBS?

DBS, dried blood spots; HEPA, high-efficiency particulate air; HVAC, heating ventilation and air conditioning.

#### Engineering

Biological safety cabinets are a critical primary containment device. There is little evidence regarding the optimal frequency of biosafety cabinets and heating ventilation and air conditioning (HVAC) high-efficiency particulate air (HEPA) filter recertification; however, annual recertification is the norm.^1^ Other issues include the lack of clear evidence for the recommendation that decontamination through gaseous fumigation be performed before recertification or other related procedures.

Facility biocontainment issues include the significant variation between leak rate measurement and airtightness between international standards and some controversy about what constitutes “adequate” air tightness. Furthermore, the impact of unscheduled laboratory HVAC system shutdown on biocontainment is not fully understood, and an appropriate operational response is required, considering the requirement for staff to exit the facility and the impact of the shutdown on the safe operation of biosafety cabinets/fume hoods. An analysis of the cause of unscheduled shutdowns including the use and abuse of emergency stop buttons should occur.

The impacts of aging on biocontainment facilities are not understood, and an improved appreciation of the agent characteristics of commonly used materials need to be assessed in terms of being able to maintain their biocontainment effectiveness as they age (e.g., silicone sealant, aluminum foam sandwich panels, double-layered plasterboard).

Showering out of high containment facilities is often required, especially where animals are housed. However, often this practice is extended to laboratories where there is no immediate risk of staff contamination. A better understanding of the evidence required for showering for specific pathogens is required. It is unclear whether the practice of showering is performed to remind staff to change their clothes before leaving the laboratory or whether it is conducted to remove pathogens from the surface of the individual's work clothes and skin.

An evidence base for showering out of a biocontainment facility significantly reduces initial costs during construction and ongoing operation and maintenance costs. Changes to showering out requirements would reduce energy costs and promote water conservation as part of operational and environmental sustainability.^2^

There is often conjecture about the correct order to remove PPE as there is inconsistency in the standard operating procedure depending on the type and requirement of the PPE and activities in question. A solid evidence base would clarify this matter. Detailed studies to determine the most effective methodologies for the reuse and/or decontamination of single-use PPE, such as N95 respirators and reusable elastomeric masks, have proven critical during the COVID-19 pandemic^3^; however, there remains a significant gap in the knowledge in this crucial area. Also, more robust scientific evidence regarding the selection, decontamination, and possible reuse of gloves and coveralls would be beneficial in low-resource settings.

In addition, the risks posed by new equipment such as Maldi-TOF or automated robotics systems processing large quantities of potentially infectious material are often unclear, which would also extend to decontamination procedures.

#### Decontamination, disinfection, and inactivation

There is a need to develop robust evidence base for standardized decontamination and disinfection recommendations. There is a lack of consensus regarding decontamination of surfaces and spaces. Furthermore, although decontamination and disinfection information is abundant for specific pathogens (e.g., SARS-CoV-2), there is often inadequate information for other important pathogens. Simplifying and standardizing surface and space decontamination would be enormously helpful to those practitioners in the field.

The use of nucleic acid amplification techniques has revolutionized diagnostics. Nucleic acid extraction techniques are often assumed to inactivate pathogens in clinical samples without a robust scientific base. Although a number of studies have been published,^4–6^ the efficacy of different nucleic acid extraction methods for the most pathogens has not been established. Therefore, there is a requirement for studies to determine the efficacy of various DNA/RNA extraction methods to inactivate a range of infectious agents.

#### Pathogen transmission

The COVID-19 pandemic has highlighted the review of fomites as a potential source of infection. However, for many other pathogens, there is little evidence regarding the potential for infectious transmission on fomites, especially the movement of staff and equipment. Furthermore, the potential production of aerosols while performing routine laboratory activities, especially in clinical settings, requires further investigation to determine the actual level of risk to the staff. This includes using point-of-care testing systems such as lateral flow devices and Xpert MTB/RTF when testing various types of samples that may have high infectious loads, including sputum.

#### Diagnostic and research issues

Dried blood spots provide a convenient and straightforward method of collecting blood samples onto a permanent medium, such as filter paper at remote clinical sites that can be sent to a centralized location for testing. However, there is very little evidence regarding biosafety risks associated with storage, sample transport, and sample punch collections.

### Biosafety Infrastructure Development Priorities

#### Engineering

A list of biosafety infrastructure priorities is presented in Table 3 and can be categorized as engineering, human, biosafety administration and pathogen-based issues. Sophisticated biocontainment laboratories require local technical input to service and calibrate the engineering facilities. Often the systems are bespoke and are not easily serviceable, or the equipment is unavailable should it need to be replaced. Can we develop risk-based approaches to biocontainment when working with highly transmissible and pathogenic agents? There are excellent examples from the Ebola outbreak in West Africa where flexible film isolators were used to process specimens before PCR amplification,^7^ indicating that there are viable alternatives to working safely with viral hemorrhagic fever samples in low-resource settings.

**Table 3.** Biosafety infrastructure priorities

Biosafety infrastructure priorities	
Human factor	
Training	• GMPP including regular updates on new information.
	• Awareness of “dual use” or “gain of function” research and the adverse implications of these activities
Reliability and competency	• Best practice for demonstrating or assessing human reliability including quantitative measures of reliability
	• Demonstrating the connection between reliability, quality, and biosafety
	• What is the best way to determine competency with infectious materials in the laboratory?
Cumbersome risk mitigation	• How to work safely—maybe the implementation of risk mitigation strategies makes matters worse as it does not allow easy working
Workforce development	• Development of biosafety as a vocation supported by locally available and relevant training.
Lack of biocontainment engineers	• Train the next generation of biocontainment engineers that will be able to provide sustainability to these facilities as well as to advise on requirements for future facilities
Biosafety administration	
Laboratory-acquired infection and escapes	• Development and implementation of a global laboratory-acquired infection or laboratory escape reporting system.
Registration of laboratories	• Regulation of laboratories worldwide especially those containing highly pathogenic agents
IBCs	• Develop a culture of IBCs to regulate and review biosafety matters within institutions.
Biosecurity and repositories	• Ensure there is a standardized and regulated method of storing pathogens using basic biorepository principles.
Pathogen-based issues	
Pathogen risk groups	• Opportunity to review the value of pathogen risk groups

GMPP, good microbiological practices and procedures; IBCs, institutional biosafety committees.

It should be recognized that often the building of the high containment laboratory, while being a source of great national or regional pride, and often, the technical aspects are secondary to this consideration. Therefore, overtime, the facility becomes a unsustainable burden on scarce financial resources, leading to increased biosafety and biocontainment risks.

#### Human factor

Developing a reliable workforce that is competent and confident to work in a laboratory with infectious materials is the cornerstone of biosafety. Training all laboratory staff in good microbiological practices and procedures, including regular updates on new information, is essential for the safe management of a laboratory. The training curriculum must include developing awareness of “dual-use” or “gain of function” research and the adverse implications of these activities. Reliability and competency go hand-in-hand in demonstrating best practices when working in a laboratory. Developing tools that can explain and quantify all aspects of this requirement would be helpful for implementation and monitoring.

Biosafety as a vocation rather than an add-on to additional duties is essential. Staff need access to relevant training, preferably as an academic program for biosafety practitioners—not just an afterthought. At present, there is limited internationally recognized formal training for biosafety professionals. Furthermore, it is of paramount importance that biosafety professionals have a background in laboratory procedures, such as those who have previously worked in virology or bacteriology laboratories. There is no substitute for experience when making judgments on risk assessments.

In addition, there is a critical lack of biocontainment engineers, especially in developing countries. Despite the proliferation of highly sophisticated biocontainment laboratories, especially in low-resource or developing country settings, there remains an acute and severe lack of biocontainment engineers to allow these facilities' sustainable management and maintenance. This problem is confounded by a lack of specific biocontainment courses for engineers, and often the only place to learn is on the job. This resource development also needs to extend to biosafety cabinet certification due to the rapid expansion of biosafety cabinets as a primary containment device.

#### Biosafety administration

Those working outside of the infectious diseases area are surprised to find very few regulations, especially those laboratories working with highly pathogenic agents. Therefore, such laboratories' registration system is essential to monitor compliance with national and international regulatory adherence and allows enforceable minimum standards for laboratory safety based on sustainable standards.

With few exceptions, only a few countries require reporting of LAIs, accidental release, or other incidents. There are formal reporting systems in Canada,^8^ the United Kingdom,^9^ and several European Union countries, and for select agents in the United States;^10^ however, most other jurisdictions have no legal requirement for LAI or infectious incident reporting. To get an accurate estimate of the actual size of issues relating to infectious incidents resulting in infections of laboratory staff or environmental contamination, formal reporting systems are required. There is a role for international organizations to serve as an “independent” or “clearinghouse” for recording such information.

Institutional biosafety committees (IBCs) play an essential role in biosafety regulation in a practical and regulatory sense. There is generally a culture of IBCs regulating and reviewing biosafety matters within well-resourced settings and more regulated institutions. Leadership often views these as necessary administrative structures and performs an essential function in research safety and community stewardship. Unfortunately, because time must be invested in describing the work and the safe measures applied for risk reduction, some researchers view IBCs as impediments to progressing research, and their value is not seen in a positive light.

The IBC should be promoted as a forum where biosafety elements and approaches can be raised and discussed without impunity to guide the end user and inform more safe practices while facilitating research projects. In less well-resourced settings or those that are not regulated, IBCs may or may not exist. When they are hastily composed and enacted, individuals without the requiste knowledge, experience, or skillsets may be selected as reviewers and the reviews are conducted in such a manner that they can be obstructive and punitive in their operation. There is an opportunity to guide and mentor new IBCs by local institutions with highly functional IBCs, local or regional biosafety associations, and international and governmental organizations.

Biosecurity and biorepositories, especially sensitive or highly infectious materials, must be regulated regarding access and accounting. There is a regulatory requirement to do so, such as in the United States, United Kingdom, and Australia; biorepository programs are regularly assessed for security breaches or discrepancies. In many countries and institutions, there are no biorepositories, and quite often, there is no systematic method of storing infectious or sensitive materials.

Pathogen risk groups have become biosafety shorthand to communicate the risk or hazard associated with a particular pathogen. The latest versions of the WOAH Terrestrial Manual^11^ and WHO Laboratory Biosafety Manual 4th edition (WHO LBM4)^12^ do not encourage the use of pathogen risk or hazard groups but instead advocate a risk-based approach to biosafety. Therefore, it is reasonable to ask—what is the value of pathogen risk groups? As scientific evidence on routes of transmission, treatment, and vaccines become available, is there a need to rely on the pathogen “classification” in conducting a biorisk assessment, or should we be putting a greater emphasis on gathering all of the pertinent information on the pathogen, procedures, and exposure mitigation factors to inform a robust biorisk assessment?

## Conclusion

Establishing a solid evidence base for biosafety practices is essential to prevent the wastage of precious resources and provide a sustainable and safe environment for laboratory activities. The research priorities identified as an outcome of the WOAH BRM and identifying the critical infrastructure deficiencies offer a rationale for future projects and activities to strengthen biosafety and biocontainment when planning future projects and activities. There is an obligation on the global biosafety and research community to support these initiatives to provide a sustainable base for the future rather than perpetuate outdated and inefficient practices.
